# Comparing the efficacy and safety of bridging therapy vs. monotherapy in patients with minor stroke: a meta-analysis

**DOI:** 10.25122/jml-2024-0318

**Published:** 2025-01

**Authors:** Abdulsalam Aleid, Saud Aldanyowi, Abdulmajeed Aljabr, Sami Almalki, Awn Alessa, Mostafa Alhodibi, Mohammed Alsuwaylih, Yousef Alanazi, Abbas Almutair

**Affiliations:** 1 Department of Surgery, Medical College, King Faisal University, Ahsa, Saudi Arabia; 2 King Saud bin Abdulaziz for Health Science, College of Medicine, Riyadh, Saudi Arabia; 3 King Abdullah International Medical Research Center (KAIMRC), Riyadh, Saudi Arabia; 4 Department of Neurosurgery, King Fahad Hospital, Hofuf, Ahsa, Saudi Arabia; 5 Primary Care, Al-Ahsa Health Cluster, Ahsa, Saudi Arabia; 6 Department of Internal Medicine, Bahrain Defense Hospital, Riffa, Saudi Arabia; 7 Department of Internal Medicine, Northern Border University, Arar, Saudi Arabia; 8 Research Center, Almoosa Hospital, Ahsa, Saudi Arabia

**Keywords:** minor stroke, bridging therapy, monotherapy, intravenous thrombolysis, endovascular thrombectomy, systematic review, meta-analysis, EVT, Endovascular Thrombectomy, IVT, Intravenous Thrombolysis, NIHSS, National Institutes of Health Stroke Scale, LVO, Large Vessel Occlusion, PRISMA, Preferred Reporting Items for Systematic Reviews and Meta-Analyses, RCT, Randomized Controlled Trials, NOS, Newcastle-Ottawa Scale, mRs, Modified Rankin Score, sICH, Symptomatic Intracranial Hemorrhage, OR, Odds Ratio, CI, Confidence Interval, I^2^, Statistical Measure of Study Heterogeneity Used in Meta-Analysis, SITS-ISTR, Safe Implementation of Treatments in Stroke–International Stroke Thrombolysis Registry, GSR-ET, German Stroke Registry–Endovascular Treatment

## Abstract

The two main therapeutic approaches for stroke treatment are endovascular thrombectomy, which involves mechanically removing the thrombus, and bridging therapy, which uses intravenous thrombolytics (IVT) prior to endovascular thrombectomy (EVT). This study aimed to compare monotherapy (EVT or IVT alone) with bridging therapy (IVT+EVT) in terms of efficacy and safety outcomes in patients with minor ischemic stroke. After a thorough screening, eight studies were included for qualitative synthesis and meta-analysis, comprising a total of 3,117 patients across the treatment arms. The main outcomes of interest were the efficacy of treatment modality, the rate of intracerebral hemorrhage (ICH), and mortality. In terms of functional outcomes measured by the Modified Rankin Score (mRs) 0-1, no significant difference was observed when comparing IVT monotherapy with bridging therapy (IVT+EVT), with an odds ratio of 0.79 (*P* = 0.41). However, IVT was associated with a decreased risk of symptomatic intracranial hemorrhage (sICH) compared to bridging therapy (OR = 0.51; *P* = 0.02), while EVT was associated with an increased risk of sICH compared to bridging therapy (OR = 8.33; *P* = 0.01). Mortality rates were comparable between IVT alone compared to bridging therapy and EVT alone compared to bridging therapy (*P* = 0.14). Although both treatment modalities share similar efficacy, there was a trend in favoring bridging therapy for mortality rates, but it was not statistically significant. Future randomized controlled trials and updated systematic reviews are needed within five to ten years to increase sample sizes and potentially identify statistically significant differences in mortality and other outcomes.

## INTRODUCTION

Stroke remains one of the leading causes of mortality and long-term disability worldwide [1]. With an aging population, the global incidence of stroke is expected to increase [2,3]. Acute ischemic stroke occurs due to a thrombus formation, and the primary treatment strategies include endovascular thrombectomy (EVT), which physically removes the thrombus and bridging therapy, where intravenous thrombolytics (IVT) are administered before EVT [4]. The severity of stroke can be assessed through various methods, including the National Institutes of Health Stroke Scale (NIHSS) score [5]. According to the NIHSS, a stroke is considered if the score is below 5 [5]. Despite its classification as 'minor', about 30% of these patients experience lasting disabilities after 90 days [6,7].

Stroke centers and countries vary in how they approach the clinical management of minor ischemic strokes. Intravenous thrombolysis (IVT) remains the recommended treatment for disabling acute ischemic stroke, regardless of the NIHSS score [8]. Although large vessel occlusion (LVO) typically results in severe strokes, approximately 10–20% of patients with minor strokes have LVO due to strong collateral circulation [9,10]. Neurological deficits occur in about 20–40% of these patients, which increases their risk of a poor outcome [11-13]. The current recommendation for patients with LVO and NIHSS scores above 5 is to combine endovascular thrombectomy with IVT [14]. However, few randomized trials have included patients with NIHSS scores of 5 or less, and results from both single-center and multicenter studies have been inconclusive [15-17]. Consequently, the benefit of combination therapy versus IVT alone in these patients remains unclear. A meta-analysis by the HERMES study group found no significant advantage of EVT over standard therapy, including IVT, in patients with NIHSS scores below 10 [14,18]. Nevertheless, observational studies suggest that early thrombectomy may lead to better outcomes in mild stroke compared to optimal medical treatment followed by rescue thrombectomy in cases of deterioration [19,20]. There is also a potential for increased risk of intracerebral hemorrhage (ICH) with combined therapy. Therefore, we performed this systematic review and meta-analysis to evaluate the efficacy and safety of monotherapy (EVT or IVT) versus bridging therapy (IVT & EVT) in patients with minor ischemic stroke.

## MATERIAL AND METHODS

This systematic review and meta-analysis followed the Cochrane Handbook for Systematic Reviews and the guidelines outlined by the Preferred Reporting Items for Systematic Reviews and Meta-Analyses (PRISMA) statement. The study protocol was registered in the International Prospective Register of Systematic Reviews (ID: CRD42024548143) [21,22].

### Database searching

We systematically searched PubMed, Web of Science, Google Scholar, and Scopus for eligible articles from inception to 2023. The search strategy employed the following keywords: “Thrombolysis” AND “Thrombectomy” AND “Stroke” AND (“Minor” OR “Mild”).

### Screening process

**Figure 1:**
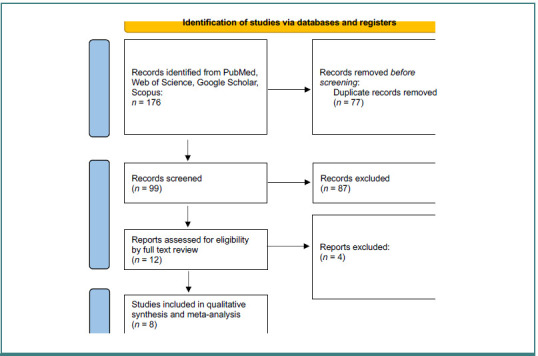
PRISMA flow diagram of screened articles

After conducting the database search, we eliminated duplicates using EndNote version 7 [23]. The remaining articles were uploaded into Rayyan software [24] to facilitate screening. Two authors independently screened the titles and abstracts to assess eligibility, followed by a full-text review of the selected studies. Any disagreements were resolved by a third author (Figure 1).

### Eligibility criteria

We applied predefined inclusion and exclusion criteria during the screening process. We included observational studies and randomized controlled trials (RCTs) that compared monotherapy, whether IVT or EVT, with bridging therapy (IVT+EVT) in patients with minor or mild ischemic stroke (NIHSS score 1–4). Studies that did not compare these two treatment strategies, which involved higher NIHSS scores, case reports, or reviews, were excluded.

### Quality assessment

For the included observational cohort studies, the New Castle Ottawa Scale (NOS) was employed to evaluate quality. Studies scoring between 0 and 3 were classified as low quality, 4–6 as moderate, and 7–9 as high quality [25].

### Data extraction

Four independent authors used Microsoft Excel to extract baseline information such as study design, sample size, age, and gender, along with outcomes like the Modified Rankin Score (mRS) 0–1, mRS 0–2, mortality, symptomatic intracranial hemorrhage (sICH), and ICH. Any discrepancies were addressed by an author not involved in the data extraction process.

### Statistical analysis

We conducted a meta-analysis using Review Manager (RevMan) version 5.4, pooling dichotomous variables to calculate odds ratios (ORs) with corresponding 95% confidence intervals (CIs). A *P* value ≤ 0.05 was considered statistically significant. Heterogeneity was assessed using the I^2^ statistic, with significance determined by the *P* value.

## RESULTS

### Database searching and screening

The database search yielded 176 articles, of which 77 were duplicates and subsequently removed. A total of 99 articles were screened by title and abstract, and 87 articles were excluded during this process. A full-text review was conducted on 12 articles, and 8 articles were included for the qualitative synthesis and meta-analysis. The total number of patients in both treatment arms across the 8 included studies was 3,117 patients (Figure 1).

### Quality assessment

According to NOS, five studies were classified as high quality, while three were considered moderate quality [17,26-32] (Table 1).

**Table 1:** Quality assessment of the included cohort studies using New Caste Ottawa Scale (NOS)

Study name	Representativeness of the exposed cohort (★)	Selection of the non exposed cohort (★)	Ascertainment of exposure (★)	Demonstration that outcome of interest was not present at start of study (★)	Comparability of cohorts on the basis of the design or analysis (max★★)	Assessment of outcome (★)	Was follow-up long enough for outcomes to occur? (★)	Adequacy of follow up of cohorts (★)	Quality level
Dobrocky *et al.*, 2021[17]	☆	-	☆		☆	☆	☆	-	Moderate
Cappellari *et al.*, 2023 [26]	☆	☆	☆	☆	☆	☆	☆	☆	High
Tu *et al.*, 2022 [27]	☆	☆	☆	☆	☆	☆	☆	☆	High
Seners *et al.*, 2020 [28]	☆	-	☆	☆	☆	☆	☆	-	Moderate
Kastrup *et al.*, 2018 [29]	☆	-	☆	☆	☆☆	☆	☆	-	High
Da Ros *et al.*, 2019 [30]	☆	☆	☆		☆☆	☆	☆	☆	High
Seners *et al.*, 2021 [31]	☆	-	☆	☆	☆	☆	☆	-	Moderate
Feil *et al.*, 2021 [32]	☆	☆	☆	☆	☆	☆	☆	☆	High

### Baseline characteristics

All included studies were cohort studies, comparing monotherapy (IVT or EVT) versus bridging therapy (IVT+EVT). The baseline characteristics of the included articles are summarized in Table 2.

**Table 2:** Baseline characteristics of the included studies

Study ID	Design	Sample size		Age, mean (SD)		Gender, male/female	
		Monotherapy	IVT+EVT	Monotherapy	IVT+EVT	Monotherapy	IVT+EVT
Dobrocky *et al.*, 2021 [17]	Cohort	84, MT: 39	85	67.7, EVT: 73.1	71.1 (30.6–97.3)	IVT: 52/32, EVT: 52/33	20/19
Cappellari *et al.*, 2023 [26]	Cohort	262	226	NR	NR	NR	NR
Tu *et al.*, 2022 [27]	Cohort	EVT:662	241	65.9(10.5)	65.7(10.8)	473/189	183/58
Seners *et al.*, 2020 [28]	Cohort	384	214	71.3 (14.3)	64.5 (16.6)	167/217	111/113
Kastrup *et al.*, 2018 [29]	Cohort	160	145	72 (12)	71 (13)	67/93	65/80
Da Ros *et al.*, 2019 [30]	Cohort	24	29	68	70 (23–92)	15/9	13/16
Seners *et al.*, 2021 [31]	Cohort	29	28	71	67 (56–75)	18/11	18/10
Feil *et al.*, 2021 [32]	Cohort	272	272	69.4 (13.7)	68.6 (14)	154/118	154/118

IVT: intravenous thrombolysis; EVT: endovascular thrombectomy; NR: not reported.

### Meta-analysis

**Figure 2:**
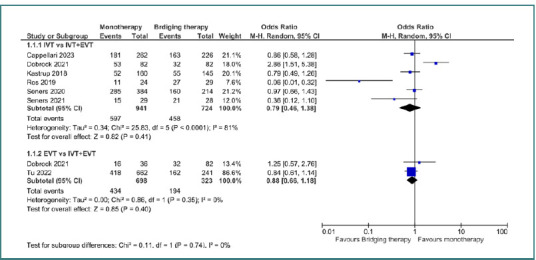
Comparison between bridging therapy and monotherapy regarding mRs 0–1

**Figure 3:**
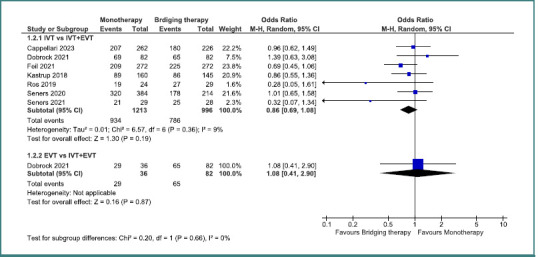
Comparison between bridging therapy and monotherapy regarding mRs 0–2

For mRS 0–1, no significant difference was found when comparing IVT monotherapy to bridging therapy (IVT+EVT), with an odds ratio of 0.79 (95% CI, 0.46–1.38; *P* = 0.41). Similarly, no significant difference was detected between EVT monotherapy and bridging therapy (OR = 0.88; 95% CI, 0.66–1.18; *P* = 0.4) (Figure 2). For mRS 0–2, no statistically significant differences emerged between IVT monotherapy and bridging therapy, with an OR of 0.86 (95% CI, 0.69–1.08; *P* = 0.19), and EVT monotherapy versus bridging therapy which yielded an OR of 1.08 (95% CI, 0.41–2.9; *P* = 0.87) (Figure 3).

**Figure 4:**
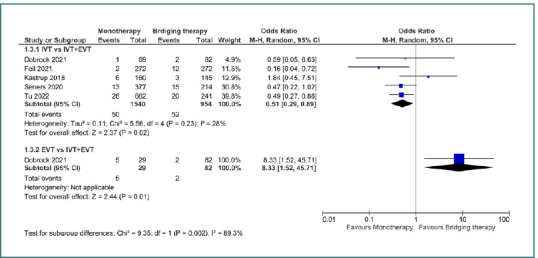
Comparison between bridging therapy and monotherapy regarding symptomatic intracranial hemorrhage

In terms of symptomatic intracerebral hemorrhage, IVT was associated with a lower risk of sICH compared to bridging therapy, with an OR of 0.51 (95% CI, 0.29–0.89; *P* = 0.02), whereas EVT was linked to a higher risk of sICH when compared to bridging therapy, with an OR of 8.33 (95% CI, 1.52–45.71; *P* = 0.01) (Figure 4).

**Figure 5:**
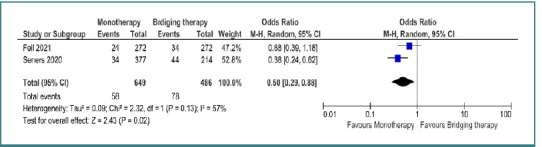
Comparison between bridging therapy and intravenous thrombolysis (IVT) regarding intracranial hemorrhage (ICH)

IVT was also associated with a reduced risk of ICH compared to bridging therapy, with an OR of 0.5 (95% CI, 0.29–0.88; *P* = 0.02) (Figure 5).

**Figure 6:**
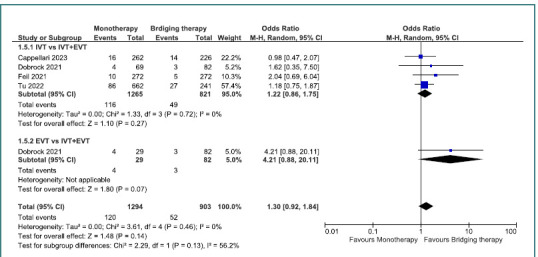
Comparison between bridging therapy and monotherapy regarding mortality

Mortality rates were similar between IVT monotherapy and bridging therapy, as well as EVT monotherapy and bridging therapy. Although there was a slight trend favoring bridging therapy, it was not statistically significant (OR =1.3; 95% CI, 0.92–1.84; *P* = 0.14) (Figure 6).

## DISCUSSION

The objective of this study was to evaluate the efficacy and safety of monotherapy with either IVT or EVT in comparison to bridging therapy (IVT+EVT) for patients with minor ischemic stroke. In terms of efficacy, the results indicated no significant differences between the treatment approaches for mRS 0-1 and mRS 0-2. However, the incidence of sICH and ICH was significantly higher in the group receiving bridging therapy compared to those treated with either IVT or EVT alone. Although EVT was associated with an elevated risk of sICH compared to bridging therapy, this finding was based on a very small sample size from a single study. The meta-analysis revealed that bridging therapy may not provide the same benefits as IVT and poses a higher risk.

The optimal treatment strategy for mild strokes remains uncertain and lacks standardization. Most patients diagnosed with mild stroke receive IVT alone, while a small subset is excluded from IVT due to their condition being perceived as too favorable to receive treatment [33]. Additionally, recent RCT meta-analyses have shown that patients with an NIHSS score below 10 do not gain significant benefit from EVT [14]. Consequently, the use of EVT in patients with LVO and NIHSS ≤ 5 has only been documented in a limited number of case series [12]. Vessel recanalization appears to play a crucial role even in minor strokes, as failure to achieve acute recanalization may result in approximately one-third of minor stroke patients being unable to walk independently at hospital discharge and facing a higher likelihood of neurological decline and poor outcomes at the 90-day follow-up [34-40].

Feil *et al*. [32] analyzed data from patients enrolled between June 2015 and December 2019 in the Safe Implementation of Treatments in Stroke–International Stroke Thrombolysis Registry (SITS-ISTR) and the German Stroke Registry–Endovascular Treatment (GSR-ET). Their findings indicated that combining EVT with IVT did not significantly enhance functional outcomes compared to IVT alone in patients with minor strokes, specifically those with NIHSS scores ≤5. Although 81.6% of GSR-ET patients treated with EVT or IVT achieved successful reperfusion (mTICI scores 2b–3), follow-up imaging at 24 hours showed a higher point estimate of sICH in patients who underwent both EVT and IVT. Nevertheless, even when performed in extended time windows, thrombectomy was carried out safely, with favorable clinical outcomes of 64%, 75%, and 60%, respectively [15,16,41]. These retrospective single-center studies included 33 patients (NIHSS score ≤8, varying occlusion sites), 41 patients (NIHSS score ≤5, M1 occlusions), and 88 patients (NIHSS score ≤4, different occlusion sites) with LVO and mild stroke symptoms [15,16,41].

Feil *et al*. [32] further reported that patients who underwent thrombectomy had notably worse functional outcomes when comparing EVT, with or without IVT, to IVT alone. Additionally, those treated with EVT had a higher median NIHSS score at the 24-hour follow-up. Logistic regression analysis revealed that IVT, but not EVT, was a strong predictor of favorable outcomes. These results differ from earlier case series, one of which reported superior outcomes for EVT patients compared to those receiving only IVT, while another case series examined 24 IVT patients alongside 32 interventional cases (19 EVT only and 13 EVT plus IVT) [12,18,30]. In the latter study, a greater shift in NIHSS scores was observed in the group undergoing endovascular procedures compared to those receiving only medical therapy. However, the interpretation of these findings may be biased, as 40% of the thrombectomy patients were ineligible for IVT [30]. Another case series involving 32 thrombectomy patients showed a greater improvement in NIHSS scores, where 25% of those primarily managed with medical therapy did not reach functional independence at follow-up [12]. In a study of 169 patients with M2 occlusion and mild stroke symptoms, no significant difference in favorable outcomes was found among those treated with IVT alone, EVT alone, or a combination of EVT and IVT. However, when analyzing only patients treated after 2015, the shift in mRS scores was significantly better in the EVT group compared to the IVT-only group [17]. Another study involving 96 patients with mild stroke found no difference in favorable clinical outcomes between the IVT group and those receiving standard medical care, although early neurological improvement was observed in IVT patients [42].

A study based on the Swiss Stroke Registry indicated that patients with mild acute ischemic stroke and LVO who underwent either IVT or EVT achieved favorable functional outcomes at three months [43]. However, further research is required to clarify the necessity of both IVT and EVT in patients with acute LVO stroke. A meta-analysis of individual patient data from five randomized trials demonstrated that EVT was more effective than standard medical treatment in cases of acute ischemic stroke caused by proximal anterior circulation artery occlusion [14]. However, the SKIP Randomized Clinical Trial did not show functional differences between the EVT and bridging groups [44]. Subsequent trials suggested that EVT alone might yield similar results to bridging therapy for patients with acute ischemic stroke due to major artery occlusions [45-47]. Interestingly, improved functional outcomes were observed in patients with large vessel occlusion stroke who received adjunct intra-arterial thrombolysis after a successful angiographic thrombectomy [48]. Moreover, findings from the Italian registry on endovascular treatment for acute stroke suggest that bridging therapy may reduce the risk of death or severe disability three months after a stroke, particularly in cases of major artery occlusion [49]. A meta-analysis involving three RCTs and six observational studies concluded that direct EVT might be as effective as bridging therapy, with a lower likelihood of intracerebral hemorrhage (ICH) and clot migration in patients with acute ischemic stroke [50]. Likewise, another meta-analysis of five observational studies reported that bridging therapy and EVT might be equally effective in managing acute anterior circulation strokes [51]. In contrast, a single-center retrospective study of 90 consecutive patients found that bridging therapy was associated with substantially higher direct and overall hospital costs than EVT alone, without demonstrating superior clinical outcomes [52]. Furthermore, Qureshi *et al*. [53] suggested that EVT alone may be more cost-effective than bridging therapy for treating acute ischemic stroke patients within 4.5 hours of symptom onset, although the study did not establish the cost-effectiveness of bridging compared to direct thrombectomy.

The limitations of the study include the fact that all the articles were cohort studies, with some having small sample sizes. Additionally, certain outcomes were based on limited sample sizes, which may have reduced the statistical power to detect significant differences. Therefore, future large-scale randomized controlled trials and an updated systematic review in the next five to ten years are recommended.

## CONCLUSION

The present results regarding efficacy outcomes revealed no statistically significant differences between the two treatment approaches in terms of mRs 0–1 and mRs 0–2. However, when bridging therapy was used instead of IVT, the safety outcomes, such as sICH and ICH, were statistically considerably higher. Furthermore, there were no discernible variations in the death rates between the two therapy modalities.
